# Surface Functionalization of Exposed Core Glass Optical Fiber for Metal Ion Sensing

**DOI:** 10.3390/s19081829

**Published:** 2019-04-17

**Authors:** Akash Bachhuka, Sabrina Heng, Krasimir Vasilev, Roman Kostecki, Andrew Abell, Heike Ebendorff-Heidepriem

**Affiliations:** 1 ARC Centre of Excellence for Nanoscale BioPhotonics, Institute for Photonics and Advanced Sensing, School of Physical Sciences, The University of Adelaide, Adelaide, SA 5005, Australia; sabrina.heng@adelaide.edu.au (S.H.); roman.kostecki@adelaide.edu.au (R.K.); andrew.abell@adelaide.edu.au (A.A.); 2 Future Industries Institute, University of South Australia, Adelaide, SA 5095, Australia; krasimir.vasilev@unisa.edu.au; 3 School of Engineering, University of South Australia, Adelaide, SA 5095, Australia

**Keywords:** surface functionalization, plasma polymerization, sensing, microstructured glass optical fibers, aluminium sensing, exposed core glass optical fibers

## Abstract

One of the biggest challenges associated with exposed core glass optical fiber-based sensing is the availability of techniques that can be used to generate reproducible, homogeneous and stable surface coating. We report a one step, solvent free method for surface functionalization of exposed core glass optical fiber that allows achieving binding of fluorophore of choice for metal ion sensing. The plasma polymerization-based method yielded a homogeneous, reproducible and stable coating, enabling high sensitivity aluminium ion sensing. The sensing platform reported in this manuscript is versatile and can be used to bind different sensing molecules opening new avenues for optical fiber-based sensing.

## 1. Introduction

Metal ions such as aluminum, lithium, calcium, zinc, iron and copper are known to have a major impact on human health [1,2] and the environment [3]. Several staining-based techniques have been developed for the detection of these metal ions. The problem with such techniques is a lack of adaptability for real time analysis [4,5]. One approach to overcome these problems uses optical fibers, as they are an attractive platform for sensing. For example, optical fiber-based metal ion sensors have been reported [6] but are limited to distal tip-based sensing [7,8]. Microstructured optical fiber (MOF)-based sensors have the potential to perform better than the traditional fluorescence or spectroscopic fiber sensors [9], as the light matter interaction can be extended along the whole length of the fiber. Suspended core microstructured optical fibers (SCFs) have a small core suspended on thin struts and are being widely used for fluorescence-based sensing [10]. SCFs have holes running along the length of fiber to provide effective refractive index for light confinement [11]. A portion of the guided light is transmitted outside of the glass and is thus available for light-matter interactions over long fiber length [12]. The holes act as tiny chambers for chemical reactions using small samples volumes [13]. SCF-based sensors for metal ion sensing have been reported [14], but a substantial obstacle for their wide use is the availability of techniques that can be employed to functionalize the surface of the core of these fibers.

In this study, a special type of SCF where the core is exposed, so called exposed core fiber (ECF) [15], was chosen as it allows easy access to the suspended core along the whole length of the fiber not possible with conventional SCFs with enclosed core, facilitating surface functionalization, reduced time of filling with analyte and thus increase in response time and enabling distributed sensing [9,16,17].

Al^3+^ was chosen as a model metal ion to investigate the capability of surface functionalized ECF for metal ion sensing as the benefits for the detection of Al^3+^ ions span from patients suffering from Alzheimer’s disease [18,19], over toxicity in plants [20,21,22] to monitoring corrosion [23,24]. Moreover, detecting and monitoring of Al^3+^ ions can minimize its impact on human health. Polyelectrolytes coated SCF have been reported to attach fluorophores for Al^3+^ ion sensing [13]. Other SCF and ECF coatings for metal ion sensing used (3-Aminopropyl) triethoxysilane (APTES) [24,25]. Moreover, Poly (methyl methacrylate) (PMMA) coated ECF has been reported for detecting Al^3+^ ions [26]. The main drawback with these coating methods is the requirement for pre-treatment of the surfaces which demand multistep processing and the use of high cost and environmentally toxic solvents. Moreover, some of these coatings lack stability in buffer solutions and also selectivity towards fluorophore binding [14]. 

**Scheme 1:**
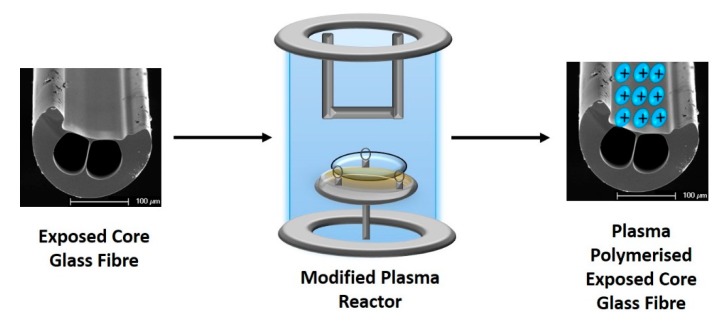
Schematic of the procedure underlying surface functionalization of microstructured optical glass fiber.

In this work, we report plasma polymer coating of an ECF (Scheme 1 and Figure S1) and demonstrated its use in Al^3+^ ion sensing. The fluorophore chosen in this work was a derivative of lumogallion with one carboxyl and two hydroxyl groups at its end [14]. The hydroxyl groups are needed for Al^3+^ ions sensing, while the carboxyl group is available for surface attachment. The chemistry and sensitivity of the fluorophore is published in our previous studies [14]. We have selected a plasma polymer with an amine group so that the carboxyl group of the fluorophore can bind to the amine group of the polymer. Plasma polymer coated ECF was immersed in a solution of an in-house synthesized derivative of lumogallion (fluorophore for Al^3+^ ions sensing) along with coupling agents (HATU and DIPEA). A schematic of the binding of fluorophore on plasma coated fiber is shown in Figure S2 of the ESI. These functionalized ECFs were then used to measure Al^3+^ ions. 

## 2. Materials and Methods

### 2.1. Chemical Synthesis and Fiber Fabrication 

Derivative of lumogallion was synthesized in house by using the procedure described elsewhere [14]. The ECFs with core diameter of 10µm was fabricated in house as has been published by Kostecki et al. [15].

### 2.2. Plasma Polymerization 

A bell chamber plasma reactor was used for surface functionalization of the ECFs. A key benefit of this technique is that it is substrate independent and no pre-modification of the surface is required [27,28,29,30]. Another advantage is that it deposits uniform and homogeneous thin films in a single step coating procedure [31,32]. In addition, it is a solvent free process, which generates no waste solvents. The ECFs were mounted in the plasma reactor using a custom-built holder with 3 pillars which aided in mounting the fiber in the chamber (Scheme 1 and Figure S1). The chamber was brought down to base pressure of 3.2 × 10^−2^ mbar. Air cleaning of the fiber was performed for 1 min by maintaining an air pressure of 1.8 × 10^−1^ mbar and igniting the plasma at a power of 50 W. Now, the chamber was brought back to base pressure. Coating of different sets of fibers with polyallylamine was performed for 2 min by maintaining a monomer pressure of 1.1 × 10^−1^ mbar at a power of 10, 25 and 40 W.

### 2.3. Immobilization of Lumogallion Derivative on ECF

Polyallylamine coated ECFs were immersed in a solution of lumogallion derivative (100 µM) along with coupling agents (HATU and DIPEA) for 3 h. Then the fibers were washed thrice with milli Q water to remove any loosely bound fluorophore. A scheme demonstrating the binding mechanism of lumogallion derivative to polyallylamine modified ECF is shown in Figure S2. 

### 2.4. Fluorescence Measurements 

Modified ECF was carefully mounted on a stage. A 5 mW laser at a wavelength of 532 nm with a 60X objective was used to couple light through its core. Fluorescence from the ECF core was collected in backward direction using the same coupling objective and recorded using a Horiba iHR550 imaging spectrometer with synapse CCD detector [26]. Autofluorescence from the plasma polymer was measured by irradiating the plasma polymerized fiber with a 532 nm laser (10 × 50 ms) while background fluorescence from the fluorophore was measured by irradiating the lumogallion functionalized fiber (in dry and wet state, i.e., without and with exposure of the functionalized fiber to water without Al^3+^ ions) with a 532 nm laser (10 × 15 ms). The background fluorescence in both wet and dry state was similar, therefore, all the background signals reported in this paper are in dry state. The presence of the azobenzene moiety in the fluorophore molecule causes unavoidable background fluorescence [14,33]. All fibers pieces used for the sensing experiments were 15 cm long and were obtained by sectioning a 45 cm long fiber into three parts of equal length. Now 2 cm of the lumogallion modified fiber was exposed to Al^3+^ ions in solution. Fluorescence from the binding of Al^3+^ ion to fluorophore functionalized fiber was then measured using the same optical setup as depicted in Figure S3. Finally, signal-to-background ratio was obtained by dividing the fluorescence counts from the binding of Al^3+^ ions to the background fluorescence from the fluorophore.

## 3. Results

**Figure 1:**
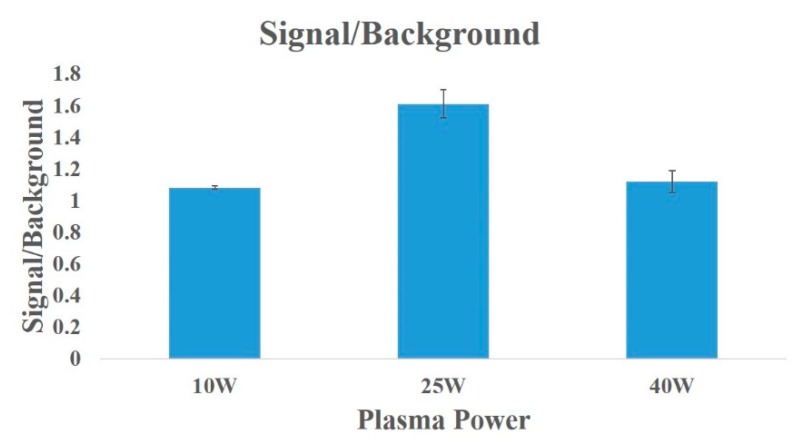
Signal-to-background ratio of surface functionalized fiber prepared with increasing plasma power. Signal-to-background ratio was obtained by dividing the fluorescence counts from the functionalized fiber exposed to aluminium ions vs the functionalized fiber by itself.

We commenced with investigating the plasma process to identify optimized plasma conditions. Plasma polymerization with increasing plasma power i.e., 10 W, 25 W and 40 W was employed to coat allylamine on the ECF. Lumogallion derivative was attached on the fibers coated with these different conditions by immersing these fibers in a solution of Lumogallion derivative. Finally, Al^3+^ ions were exposed to the fluorophore modified ECF and the fluorescence intensity was measured by using the optical setup shown in Figure S3. As shown in Figure 1, 25 W showed higher signal-to-background ratio in comparison to 10 W and 40 W. A lower plasma power (10 W), results in less fragmentation of the original structure of the precursor which results in greater retention of amine functional groups on the surface [34]. These can then react with the hydroxyl group on the fluorophore rather than the desired carboxylic acid. More binding sites for fluorophores are thus available, reducing the number of hydroxyl groups available for binding Al^3+^ ions and thus decreasing the fluorescence signal. However, such films deposited at lower plasma power are also known to have lower degree of crosslinking leading to dissolution in aqueous solvents resulting in lower efficacy for binding fluorophores [35]. By contrast, higher plasma power (40 W), results in greater fragmentation of the monomer which results in less amine functional groups on the surface of the fiber [36]. A lower density of amine groups is then available for binding to the fluorophore, leading to lower density of fluorophore molecules available for binding to Al^3+^ and thus ultimately lower fluorescence signal. 

Therefore, we hypothesize that there is a balance between low power (10 W) and high power (40 W) i.e., at a medium power of 25 W that increases the probability of fluorophore attachment in the desired orientation to the surface. Based on these results, all fibers for sensing were surface functionalized at a power of 25 W. 

**Figure 2:**
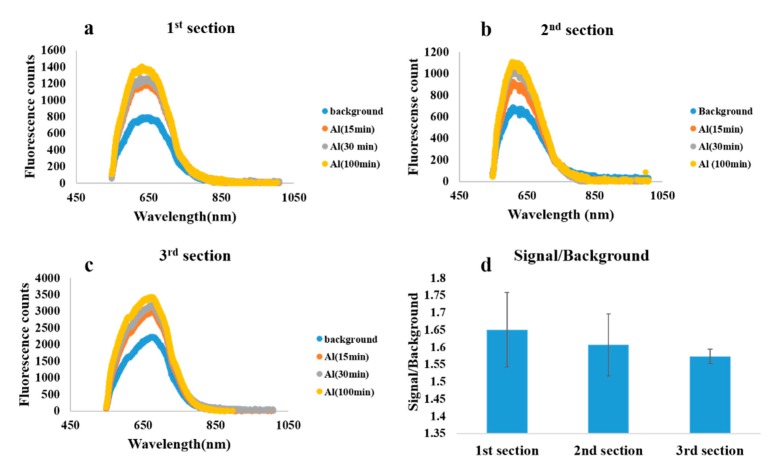
Fluorescence counts for a surface functionalized ECF (at plasma power of 25 W)-based aluminium sensor. (**a**–**c**) Each plot corresponds to a different section of the same fiber. Blue curves correspond to background fluorescence from the fluorophore-coated fiber. Orange, grey and yellow curves were recorded at 15, 30 and 100 min after exposing the fiber to 1 mM aluminium ion solution. (**d**) Bar plot corresponds to the average of signal-to-background ratio recorded at 100 min for sections (1st, 2nd and 3rd) of 3 fibers each.

The homogeneity, reproducibility and sensitivity of plasma-coated fibers was measured by sectioning a 45 cm long fiber into three parts of equal lengths (15 cm each). All the fibers for these experiments were coated at a plasma power of 25 W, as the impact of plasma power showed that 25 W has best signal-to-background ratio as can be seen in Figure 1. First, the background fluorescence (in dry state) of the functionalized fiber section was measured (blue curves in Figure 2). In the next step, each fiber section was exposed to a 1mM solution of Al^3+^ ions in water and the fluorescence was measured. The signal-to-background ratio values appear to be different for the 3 sections (1st, 2nd and 3rd section of the fiber) but they all fall within the error bar. For all three fiber sections, the average value of signal-to-background ratio was observed to be 1.6 ± 0.15 as shown in Figure 2d. Fluorescence counts after exposure to Al^3+^ ions for 15 min showed a 1.5-fold increase in fluorescence while a 1.75-fold increase was observed after 100 min of exposure compared to background fluorescence (Figure 2a–c). The Al^3+^ ion sensitivity reported in this paper (1 mM) using plasma polymer functionalized ECF is ten times higher than the sensitivity (10 mM) published by Warren-Smith et al. for polyelectrolyte functionalized SCF using the same fluorophore [14]. Both fibers have cores suspended in air by thin struts. The SCF (with enclosed core) in [14] has a considerably smaller core diameter of 1.7µm compared with the ECF (with exposed suspended core) used in this work (10 µm core diameter). The small core of the SCF in [14] offers higher sensitivity due to larger light-matter overlap compared to larger core of our ECF [16]. Therefore, the SCF in [14] should have a higher signal then our ECF, but the opposite result is observed, indicating that the higher sensitivity of our ECF is caused by plasma polymer functionalization compared to the polyelectrolytes functionalization reported in [14]. This demonstrates that the sensitivity of the same fluorophore can be controlled by choosing different surface functionalization techniques. To confirm reproducibility, homogeneity and sensitivity we repeated the experiments in triplicates of triplicates. The signal-to-background ratio remained the same across different sets of fibers. 

**Figure 3:**
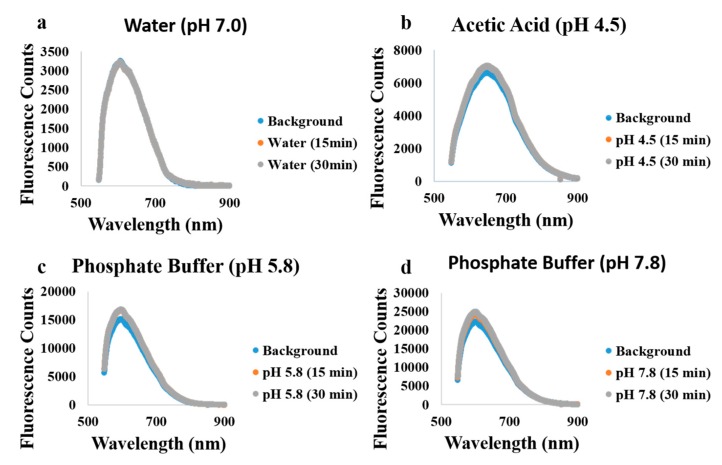
Fluorescence counts for a plasma polymerized ECF. Blue curve corresponds to auto fluorescence from the plasma polymer coating. Orange and grey curves correspond to auto fluorescence after exposure to different buffer solutions for 15 and 30 min respectively.

Finally, plasma polymerized fibers were exposed to a buffer solution of sodium acetate (pH 4.5), water (pH 7.0) and phosphate buffer (pH 5.8 and 7.8) in order to determine chemical stability of the plasma polymer coating. Autofluorescence of the plasma polymer coating was used to study the stability of these polymers before and after exposure to different buffer solutions. As shown in Figure 3, no change in autofluorescence was observed before and after exposure of the polymer coating to different buffer solutions. This demonstrates that the polymer coatings are stable in different buffer solutions.

## 4. Discussion and Conclusions

In summary, we report a novel, solvent-free technique for generating homogeneous, reproducible and stable coatings on exposed core fiber which can be further used for immobilizing sensor molecules for sensing ions. We demonstrate the feasibility of the technique by employing fluorophore specific to Al^3+^ ions. The activity of the fluorophore is controlled by varying the RF power of the plasma generator. 

We have demonstrated the reproducibility and homogeneity of the coatings across different sections of the same fiber. Moreover, an order of magnitude improvement in sensitivity of the same fluorophore attached to MOF with different surface functionalization technique was reported in comparison to the previously published studies. Furthermore, the stability of the coatings in different buffer solutions (sodium acetate buffer, water and phosphate buffer) ranging from pH 4.5 to 7.8 was demonstrated. The technology presented in this manuscript has enormous potential for applications in various fields such as metal ion sensing and bio-sensing. Considering the vast variety of plasma polymerized precursors, the physical and chemical properties of the coatings can be easily tuned to generate polymers specific to different fluorophores and antibodies. These can further be employed to capture or to detect different entities expanding its capabilities in a broad range of fields such as biological systems, environment and aviation.

## Supplementary Materials

The following are available online at https://www.mdpi.com/1424-8220/19/8/1829/s1, Figure S1: Schematic of plasma reactor and custom- built fiber holder, Figure S2: Schematic showing the coupling of lumogallion derivative with plasma polymerized ECF, Figure S3: Schematic of optical setup.

## References

[1] Chasapis C.T., Loutsidou A.C., Spiliopoulou C.A., Stefanidou M.E. (2012). Zinc and Human Health: An Update. Arch. Toxicol..

[2] Pérez-Granados A.M., Vaquero M.P. (2002). Silicon, Aluminium, Arsenic and Lithium: Essentiality and Human Health Implications. J. Nutr. Health Aging.

[3] Ščančar J., Milačič R. (2006). Aluminium Speciation in Environmental Samples: A Review. Anal. Bioanal. Chem..

[4] House E., Collingwood J., Khan A., Korchazkina O., Berthon G., Exley C. (2004). Aluminium, Iron, Zinc and Copper Influence the In Vitro Formation of Amyloid Fibrils of Aβ_ {42} in a Manner which may have Consequences for Metal Chelation Therapy in Alzheimer’s Disease. J. Alzheimers Dis..

[5] Chambers B., Taylor S. (2007). The High Throughput Assessment of Aluminium Alloy Corrosion Using Fluorometric Methods. Part I–Development of a Fluorometric Method to Quantify Aluminium Ion Concentration. Corros. Sci..

[6] Potyrailo R.A., Hobbs S.E., Hieftje G.M. (1998). Near-Ultraviolet Evanescent-Wave Absorption Sensor Based on a Multimode Optical Fiber. Anal. Chem..

[7] Ahmad M., Narayanaswamy R. (2002). Optical Fibre Al (III) Sensor Based on Solid Surface Fluorescence Measurement. Sens. Actuators B Chem..

[8] McAdam G., Newman P., McKenzie I., Davis C., Hinton B. (2005). Fiber Optic Sensors for Detection of Corrosion within Aircraft. Struct. Health Monit..

[9] Warren-Smith S.C., Ebendorff-Heidepriem H., Foo T.C., Moore R., Davis C., Monro T.M. (2009). Exposed-Core Microstructured Optical Fibers for Real-Time Fluorescence Sensing. Opt. Express.

[10] Schartner E.P., Tsiminis G., François A., Kostecki R., Warren-Smith S.C., Nguyen L.V., Heng S., Reynolds T., Klantsataya E., Rowland K.J. (2015). Taming the light in microstructured optical fibers for sensing. Int. J. Appl. Glass Sci..

[11] Knight J.C., Birks T.A., Russell P.S.J., Atkin D.M. (1996). All-Silica Single-Mode Optical Fiber with Photonic Crystal Cladding. Opt. Lett..

[12] Monro T.M., Richardson D., Bennett P. (1999). Developing Holey Fibres for Evanescent Field Devices. Electron. Lett..

[13] Monro T.M., Belardi W., Furusawa K., Baggett J.C., Broderick N., Richardson D. (2001). Sensing with Microstructured Optical Fibres. Meas. Sci. Technol..

[14] Warren-Smith S.C., Heng S., Ebendorff-Heidepriem H., Abell A.D., Monro T.M. (2011). Fluorescence-Based Aluminum Ion Sensing Using a Surface-Functionalized Microstructured Optical Fiber. Langmuir.

[15] Kostecki R., Ebendorff-Heidepriem H., Davis C., McAdam G., Warren-Smith S.C., Monro T.M. (2012). Silica Exposed-Core Microstructured Optical Fibers. Opt. Mater. Express.

[16] Kostecki R., Ebendorff-Heidepriem H., Warren-Smith S.C., McAdam G., Davis C., Monro T.M. (2013). Optical Fibres for Distributed Corrosion Sensing—Architecture and Characterisation. Key Eng. Mater..

[17] Li J., Ebendorff-Heidepriem H., Gibson B.C., Greentree A.D., Hutchinson M.R., Jia P., Kostecki R., Liu G., Orth A., Ploschner M. (2018). Perspective: Biomedical sensing and imaging with optical fibers—Innovation through convergence of science disciplines. APL Photonics.

[18] Campbell A. (2002). The Potential Role of Aluminium in Alzheimer’s Disease. Nephrol. Dial. Transplant..

[19] McLachlan D.C., Lukiw W., Kruck T. (1989). New Evidence for an Active Role of Aluminum in Alzheimer’s Disease. Can. J. Neurol. Sci..

[20] Matsumoto H. (2000). Cell Biology of Aluminum Toxicity and Tolerance in Higher Plants. Int. Rev. Cytol..

[21] Liu J., Piñeros M.A., Kochian L.V. (2014). The Role of Aluminum Sensing and Signaling in Plant Aluminum Resistance. J. Integr. Plant Biol..

[22] Gupta N., Gaurav S.S., Kumar A. (2013). Molecular Basis of Aluminium Toxicity in Plants: A Review. Am. J. Plant Sci..

[23] Shrestha B.R., Hu Q., Baimpos T., Kristiansen K., Israelachvili J.N., Valtiner M. (2015). Real-Time Monitoring of Aluminum Crevice Corrosion and Its Inhibition by Vanadates with Multiple Beam Interferometry in a Surface Forces Apparatus. J. Electrochem. Soc..

[24] Kostecki R., Ebendorff-Heidepriem H., Davis C., McAdam G., Wang T., Monro T. Fiber Optic Approach for Detecting Corrosion. Proceedings of the SPIE Smart Structures and Materials + Nondestructive Evaluation and Health Monitoring.

[25] Heng S., McDevitt C.A., Kostecki R., Morey J.R., Eijkelkamp B.A., Ebendorff-Heidepriem H., Monro T.M., Abell A.D. (2016). Microstructured Optical Fiber-based Biosensors: Reversible and Nanoliter-Scale Measurement of Zinc Ions. ACS Appl. Mater. Interfaces.

[26] Kostecki R., Ebendorff-Heidepriem H., Afshar S.V., McAdam G., Davis C., Monro T.M. (2014). Novel Polymer Functionalization Method for Exposed-Core Optical Fiber. Opt. Mater. Express.

[27] Michelmore A., Martinek P., Sah V., Short R.D., Vasilev K. (2011). Surface Morphology in the Early Stages of Plasma Polymer Film Growth from Amine-Containing Monomers. Plasma Processes Polym..

[28] Vasilev K., Michelmore A., Griesser H.J., Short R.D. (2009). Substrate influence on the initial growth phase of plasma-deposited polymer films. Chem. Commun..

[29] Von Keudell A., Benedikt J. (2010). A physicist’s perspective on “views on macroscopic kinetics of plasma polymerisation”. Plasma Processes Polym..

[30] Gleason K.K. (2010). A chemical engineering perspective on “Views on macroscopic kinetics of plasma polymerisation”. Plasma Processes Polym..

[31] Rinsch C.L., Chen X., Panchalingam V., Eberhart R.C., Wang J.-H., Timmons R.B. (1996). Pulsed radio frequency plasma polymerization of allyl alcohol: Controlled deposition of surface hydroxyl groups. Langmuir.

[32] Michelmore A., Steele D.A., Whittle J.D., Bradley J.W., Short R.D. (2013). Nanoscale deposition of chemically functionalised films via plasma polymerisation. RSC Adv..

[33] Stubing D.B., Heng S., Monro T.M., Abell A.D. (2017). A comparative study of the fluorescence and photostability of common photoswitches in microstructured optical fibre. Sens. Actuators B Chem..

[34] Ruiz J.C., Taheri S., Michelmore A., Robinson D.E., Short R.D., Vasilev K., Förch R. (2014). Approaches to Quantify Amine Groups in the Presence of Hydroxyl Functional Groups in Plasma Polymerized Thin Films. Plasma Processes Polym..

[35] Vasilev K., Britcher L., Casanal A., Griesser H.J. (2008). Solvent-Induced Porosity in Ultrathin Amine Plasma Polymer Coatings. J. Phys. Chem. B.

[36] Vasilev K., Michelmore A., Martinek P., Chan J., Sah V., Griesser H.J., Short R.D. (2010). Early Stages of Growth of Plasma Polymer Coatings Deposited from Nitrogen-and Oxygen-Containing Monomers. Plasma Processes Polym..

