# Supplementary material for: Surface Functionalization of Exposed Core Glass Optical Fiber for Metal Ion Sensing
Source: Sensors (Basel). 2019 Apr 17;19(8):1829. doi: 10.3390/s19081829 (PMC6515075; doi:10.3390/s19081829)
Supplement: Supplementary file 1 [file sensors-19-01829-s001.pdf]

Supporting Information

Surface Functionalization of Exposed Core Glass Optical Fiber for Metal Ion Sensing

Akash Bachhuka [1,*], Sabrina Heng [1], Krasimir Vasilev [2,3], Roman Kostecki [1], Andrew Abell [1] and Heike Ebendorff-Heidepriem [1,*]

[1] ARC Centre of Excellence for Nanoscale BioPhotonics, Institute for Photonics and Advanced Sensing, School of Physical Sciences, The University of Adelaide, Adelaide, SA, 5005, Australia; sabrina.heng@adelaide.edu.au (S.H.); roman.kostecki@adelaide.edu.au (R.K.); andrew.abell@adelaide.edu.au (A.A.)

[2] Future Industries Institute, University of South Australia, Krasimir Vasilev SA 5095, Australia; krasimir.vasilev@unisa.edu.au

[3] School of Engineering, University of South Australia, Krasimir Vasilev SA 5095, Australia;

* Correspondence: akash.bachhuka@adelaide.edu.au (A.B.); heike.ebendorff@adelaide.edu.au (H.E.-H.)

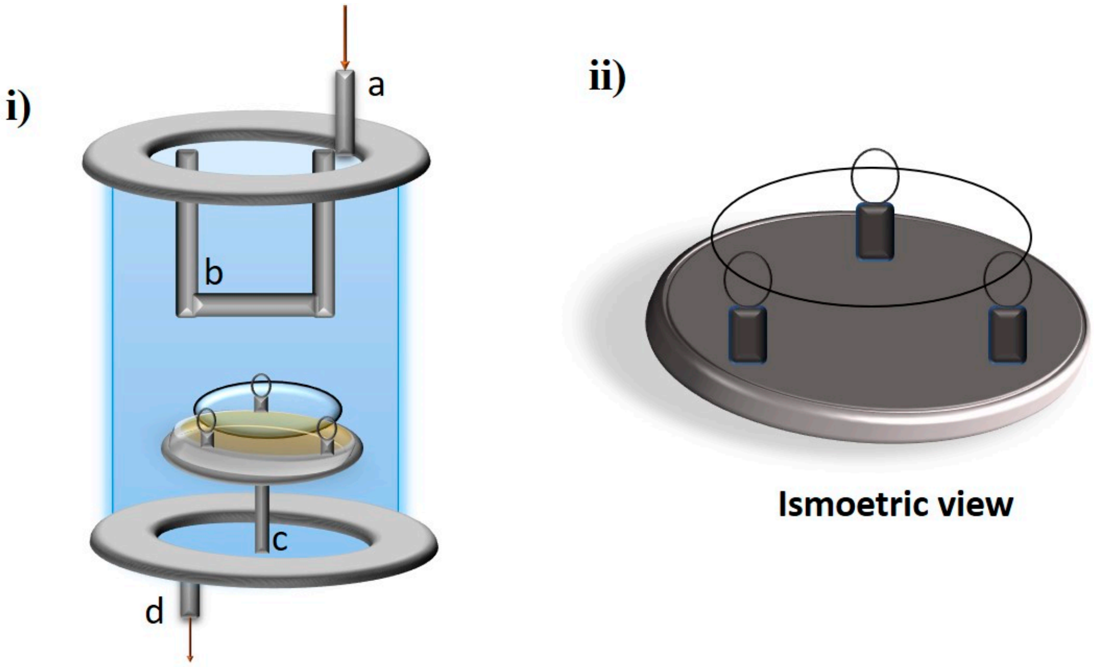

Figure S1. i) Schematic of plasma reactor with an additional holder for suspending fiber a) monomer inlet b) RF cathode c) RF anode with attached holder d) monomer outlet ii) Schematic of custom-built fiber holder (isometric view).

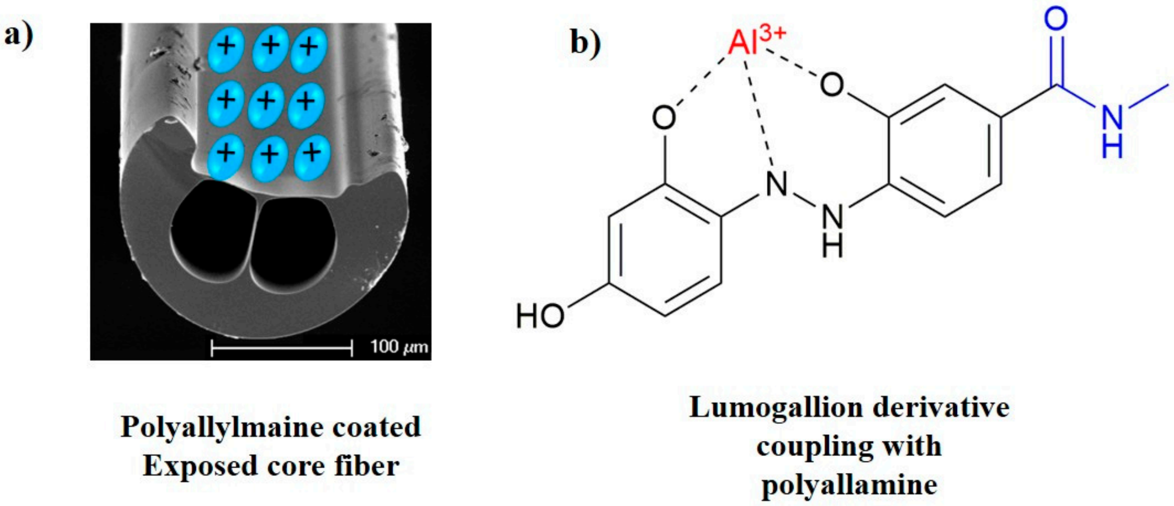

Figure S2. a) Schematic showing exposed core fiber (ECF) coated with plasma polymerized allylamine. b) Simplified version of lumogallion derivative coupling with plasma polymerized ECF. (Adapted from Reference 14)

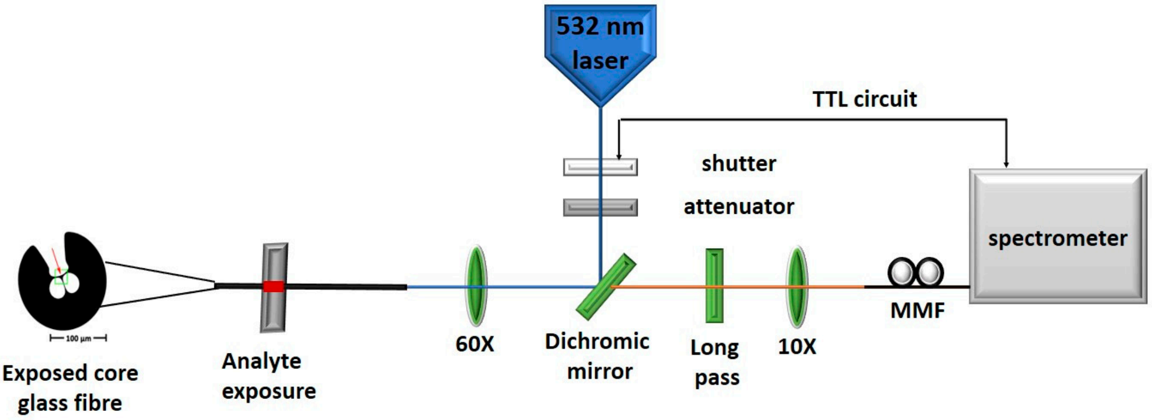

Figure S3. Schematic of the optical setup used to measure fluorescence from functionalized exposed core fiber (ECF) when exposed with the Al^{3+} ions. A 532 nm laser was used for alignment and for all measurements. The fibers were exposed to Al^{3+} ions by immersing a part of the fiber, as is demonstrated in the above figure.
